# Comparison of Salivary Opiorphin in Burning Mouth Syndrome and Healthy Subjects and Its Correlation With Psychiatric Disorders

**DOI:** 10.1002/cre2.934

**Published:** 2024-12-01

**Authors:** Zohreh Dalirsani, Maryam Amirchaghmaghi, Mahshid Malakuti Semnani, Mahdi Talebi, Seyed Isaac Hashemy, Mohammad Taghi Shakeri, Ava Taghizadeh

**Affiliations:** ^1^ Oral and Maxillofacial Diseases Research Center Mashhad University of Medical Sciences Mashhad Iran; ^2^ Department of Oral and Maxillofacial Medicine, School of Dentistry Khorasan Shomali University of Medical Sciences Bojnord Iran; ^3^ Department of Community and Family Medicine, Faculty of Medicine Mashhad University of Medical Sciences Mashhad Iran; ^4^ Surgical Oncology Research Center Mashhad University of Medical Sciences Mashhad Iran; ^5^ Social Determinants of Health Research Center Mashhad University of Medical Sciences Mashhad Iran; ^6^ Department of Oral and Maxillofacial Medicine, School of Dentistry Tehran University of Medical Sciences Tehran Iran

**Keywords:** anxiety, burning mouth syndrome, depression, opiorphin, saliva

## Abstract

**Objectives:**

Burning mouth syndrome (BMS) is a chronic disease in which the patient experiences a burning sensation in the oral mucosa without any pathological cause. Opiorphin is a pentapeptide that prevents pain and can have an antidepressant effect. The aim of this study was to evaluate salivary opiorphin concentration in BMS and healthy subjects. In addition, opiorphin concentration in BMS patients before and after psychiatric treatment was compared and the association between burning severity and psychiatric scores with salivary opiorphin concentration was examined.

**Materials and Methods:**

This cross‐sectional study included 28 BMS patients and 40 healthy individuals referred to the Department of Oral and Maxillofacial Medicine, Faculty of Dentistry, Mashhad, from September 2018 to August 2019. Patients with proven disease based on clinical and psychiatric examinations were included in the study. Unstimulated salivary opiorphin levels were assessed by ELISA. Statistical analysis was performed using SPSS version 24.

**Results:**

Salivary opiorphin levels were significantly higher in BMS patients (2.16 ± 0.30 ng/mL) than in healthy subjects (1.80 ± 0.36 ng/mL) (*p* < 0.001). Opiorphin levels in BMS patients increased insignificantly after psychiatric therapy (*p* = 0.212). In addition, there was no significant association between opiorphin levels and age, gender, menopause, burning severity, anxiety, and depression status.

**Conclusions:**

The opiorphin concentration in saliva is higher in BMS patients than in healthy subjects. Most BMS patients also suffer from anxiety and depression.

## 1 Introduction

Burning mouth syndrome (BMS) is a chronic intraoral burning sensation that lasts for more than 2 h per day for at least 3 months (Headache Classification Committee of the International Headache Society (IHS) 2018). The burning sensation is generally bilateral and the patient may experience oral dryness, dysesthesia, and taste alteration (Calabria et al. 2024; Rabiei, Leili, and Alizadeh 2018). According to a systematic review, the global prevalence of BMS in the general population is about 1.73%, while its prevalence in clinical patients increases to approximately 8%. This condition is mainly reported in middle‐aged and elderly women, with a female‐to‐male ratio of 3:1 (Wu et al. 2022).

There are gender differences in the perception of pain. Women report chronic pain more often than men. In addition, they have lower pain thresholds and lower pain tolerance. Therefore, women generally experience more unpleasant and intense pain. It appears that genetics and hormones play a role in gender differences in pain perception and the prevalence of BMS (Hairi et al. 2013, Mills, Nicolson, and Smith 2019, Calabria et al. 2024). Studies show that there is no local or systemic disease that explains the burning sensation in BMS patients (Gurvits, 2013, Salarić, Sabalić, and Alajbeg 2017) and that patients complain of chronic burning sensation in the mouth even though the oral mucosa appears normal (2018).

However, the cause of disease is still unknown, whereas its pathogenesis is influenced by psychological factors and peripheral and central neuropathy (Feller et al. 2017). Studies suggest a connection between psychosocial and/or psychiatric disorders with this disease; it has been found that most BMS patients suffer from anxiety and/or depression (Rabiei, Leili, and Alizadeh 2018). In many cases, BMS is misdiagnosed and inappropriate treatments could increase patients' anxiety and consequently increase the severity of burning sensation. Therefore, the diagnosis and treatment of BMS require the involvement of various specialists (Sun et al. 2013).

To the best of our knowledge, no specific paraclinical parameters for BMS have been found and the diagnosis is based solely on clinical findings (Baharvand, Rafiean, and Bakhtiyari 2010). One of the factors that has been considered in recent years to assess chronic pain is opiorphin. Opiorphin is a pentapeptide in human saliva and, as a natural analgesic, directly prevents the breakdown of enkephalins (Boucher et al. 2017, Salarić, Sabalić, and Alajbeg 2017). Furthermore, opiorphin may indirectly contribute to pain perception in BMS patients by altering the level of the pro‐inflammatory peptide of substance‐p in saliva (Boucher et al. 2017, Ozdogan et al. 2019). Opiorphin also exerts an antidepressant effect via the opioid receptor γ and µ and modulates the concentration of enkephalin (Javelot et al. 2010, Boucher et al. 2017, Salarić, Sabalić, and Alajbeg 2017).

Given the strong association between BMS and psychiatric disorders (Rabiei, Leili, and Alizadeh 2018) as well as the analgesic and antidepressant effects of opiorphin (Boucher et al. 2017, Ozdogan et al. 2019), the aim of this study was to examine the association between opiorphin levels and pain severity and psychiatric scores. We compared the salivary opiorphin levels of BMS patients, as a marker, with those of healthy individuals. Since there are no specific diagnostic methods to determine the rate of improvement of patients after treatment, salivary levels of opiorphin before and after psychiatric treatment were compared. We wanted to determine whether this marker could be used to understand the pathogenesis of this disease and monitor response to treatment.

## 2 Materials and Methods

### 2.1 Participants

This cross‐sectional study was conducted on patients referred to the Department of Oral and Maxillofacial Diseases, Faculty of Dentistry, Mashhad University of Medical Sciences, Iran, from September 2018 to August 2019. Since this was the first study to investigate the impact of psychiatric treatment on the severity of BMS, all patients referred within this interval and who fulfilled the inclusion criteria participated in the study. All participants signed informed consent forms. The study protocol was approved by the Ethics Committee of Mashhad University of Medical Sciences under the code IR.MUMS.DENTISTRY.REC.1397.045.

BMS patients were identified using diagnostic criteria and an interview with a psychiatrist. The diagnostic criteria were based on the guidelines of the International Classification of Headache Disorders (2018). Inclusion criteria for the BMS patients were age between 18 and 65 years, normal oral mucosa, burning sensation during the day for 3–6 months, and no treatment in the last 4 weeks. In addition, 40 healthy subjects referred to our dental clinic and who fulfilled the inclusion criteria were selected as controls. Therefore, the two groups were almost similar in terms of residential area and socioeconomic status. Inclusion criteria for the control group were age between 18 and 65 years, no oral or dental pain and no chronic pain, systemic disease, or drug consumption in the last 4 weeks (Sardella et al. 1999).

Exclusion criteria for both groups were systemic or topical factors that could cause burning sensation, including diabetes, anemia, vitamin B_12_ or folic acid deficiency, Sjögren's syndrome or other hyposalivation states, smoking (Gao et al. 2009), or pathological changes in the oral cavity such as candidiasis, lichen planus, severe mental disorders such as insanity, suicidal attempt, admission to a mental hospital (Sardella et al. 1999, Sardella et al. 2006), taking medications that affect opiorphin levels (since opiorphin is not recognized as a medication, drugs that interact with opioids, such as analgesics, were considered), and taking antidepressants, anxiolytics, or other medications that have side effects such as dry mouth or burning sensation.

Additional exclusion criteria for the control group were a diagnosis of anxiety, depression, or psychiatric disorders based on the Hamilton questionnaire (for anxiety scores higher than 18 and/or depression scores higher than 6). This process was confirmed by psychiatrist.Also, bloodborne infectious diseases transmitted through saliva such as HIV, HTLV, and hepatitis B, C, and D were excluded (Flowchart 1).

**Figure 1:**
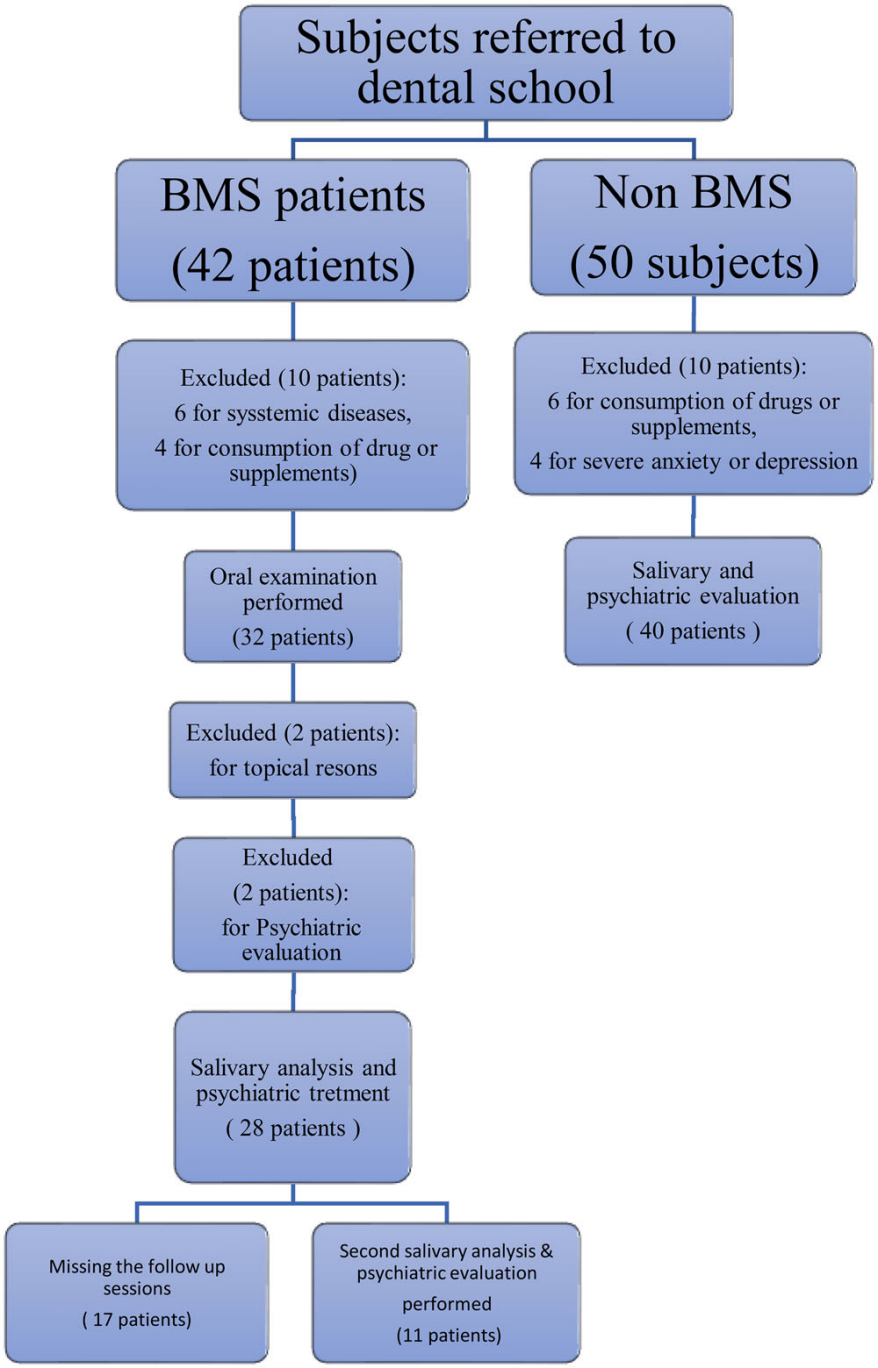
Flowchart for subject selection.

The selection of subjects for both groups is shown in Figure 1. Data were collected from the patients' medical records, laboratory tests, and/or interviews.

### 2.2 Saliva Analysis

Saliva collection and oral examination were performed by an oral medicine specialist. Unstimulated saliva was collected from both groups from spit between 9 and 11 a.m. The subjects were asked not to eat, drink, smoke, or brush their teeth for 90 min before the test (Shirzaiy et al. 2017).

The manufacturer's instructions were fully followed when carrying out the study steps. Saliva samples were immediately separated into 10 mL tubes and were kept frozen for 24 h for mucolysis, liquefaction, and homogenization. After thawing, centrifugation was performed at a speed of 2000–3000 rpm for 15 min to remove mucus. The aqueous part was stored at –20°C until all samples were collected (Dalirsani et al. 2023). The Human Opiorphin ELISA Kit (Catalog No. MBS760008, Mybiosource Co.) was used to measure opiorphin.

The Biotin double antibody sandwich kit was used to prepare the samples; 40 µL of the sample and 10 µL of the kit antibody were added to 50 µL of the streptavidin‐HRP kit and incubated for 60 min at 37°C. Next, 10 mL of buffer kit was mixed with 290 mL of water and rinsed five times. Fifty microliters of chromogen A and 50 µL of chromogen B were then added and incubated for 10 min at 37°C for color recognition. Ten minutes after adding 50 µL of stop‐kit solution to prevent further reactions, the absorption rate (OD) was measured at 450 nm (Dalirsani et al. 2023). The higher the concentration of opiorphin, the stronger the blue became. Opiorphin concentration was defined using the standard kit diagram.

### 2.3 Questionnaires

The Visual Analogue Scale (VAS) was used to assess pain severity using a 10‐point rating scale. The assessment was performed at the initial examination and repeated 4 weeks after the clinical and psychiatric examination. Response to treatment for psychiatric disorders usually begins approximately 4 weeks after therapy and is assessed by reduction in signs and symptoms using the Hamilton questionnaire and a psychiatric interview.

For the case and control groups, the Hamilton Anxiety Test (HAD‐A) and Hamilton Depression Test (HAD‐D) questionnaires were completed to assess anxiety and depression. The Hamilton Rating Scale is one of the clinical rating scales used to assess anxiety and depression. The depression questionnaire includes 21 items that measure the physical, mental, and behavioral symptoms of depression. The anxiety questionnaire consists of a 14‐item scale that assesses both physical and cognitive symptoms of anxiety. In this questionnaire, each item has five points, ranging between zero and four depending on the severity of the symptoms. The test was scored by a psychiatrist (Akhondzadeh et al. 2005). After completing the questionnaire for the case and control groups, a psychiatrist interviewed BMS patients to diagnose psychiatric disorders. Participants in the control group who were diagnosed with anxiety, depression, or other psychiatric disorders confirmed by a questionnaire and interview with the psychiatrist were excluded from the study (four subjects).

The first line of antidepressant treatment for patients was SSRIs (selective serotonin reuptake inhibitors) in combination with citalopram. Patient recovery in this study was defined as a reduction in symptoms of at least 50%, as determined by clinical interview before and after treatment. After 4 weeks, the Hamilton questionnaire was completed and a psychiatric interview and VAS assessment were performed to determine response to treatment.

### 2.4 Statistical Analysis

Data analysis was performed using SPSS version 24. After validating the normal distribution of quantitative variables using the Kolmogorov–Smirnov normality test, the independent *t*‐test was used for normally distributed data and the Mann–Whitney test was used for non‐normally distributed data. To compare and evaluate the qualitative variables between the two groups, the *χ* ^2^ and Fisher's exact tests were used. The significance level was set at 0.05.

## 3 Results

In this study, 68 participants were included based on the inclusion criteria, of whom 28 were BMS patients, including 5 (18%) men and 23 (82%) women, and 40 were controls, including 9 (23%) men and 31 (77%) women. The two groups did not differ significantly in terms of age and gender. The number of postmenopausal subjects in BMS patients (*p *> 0.05) was significantly higher than that in the control group (*p* = 0.032) (Table 1). Most of the subjects were homemakers (57.14% in the BMS group and 62.5% in the control group). In addition, most of the participants had a high school diploma (57.14% in the BMS group and 47.5% in the control group).

**Table 1:** Demographic information and psychiatric disorders of case and control subjects.

Parameter	BMS group	Control group	*p* value
Age (years),^a^ mean ± SD	52.57 ± 9.76	51.28 ± 9.85	0.595
Gender^a^			
Male, *n* (%)	5 (17.85)	9 (22.50)	0.641
Female, *n* (%)	23 (82.14)	31 (77.50)	
Postmenopause, *n* (%)^b^	17 (73.91)	14 (45.16)	0.032
Anxiety, *n* (%)^a^	28 (100)	—c	< 0.001
Depression, *n* (%)^a^	25 (89.28)	—c	< 0.001

^a^ Independent *t*‐test.

^b^ *χ* ^2^ test.

^c^ At the beginning of the study, four control participants were excluded from the study due to their anxiety scores (based on exclusion criteria).

**Figure 2:**
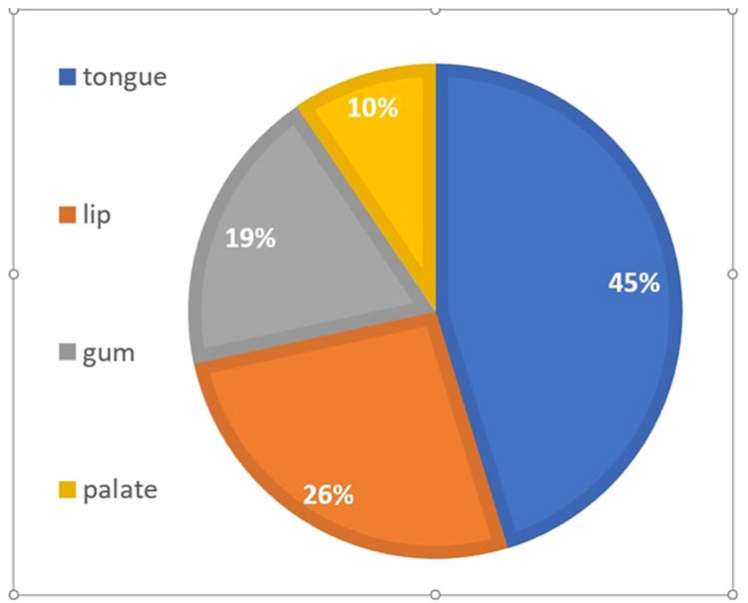
Location of burning sensation reported by BMS patients.

The duration of burning sensation in the BMS group was between 0.4 and 10 years, with a mean of 1.75 ± 2.12 years. The most common sites in the oral cavity were the tongue, lips, gums, and palate (Figure 2).

Among BMS patients, 20 had not received any treatment before entering the study, whereas eight patients had received treatments consisting of herbal medicine (four patients), analgesics (one patient), antibiotics (one patient), antifungals (one patient), and anti‐plaque mouthwash (one patient). In addition, four patients were excluded from the study because they had taken medication at the time of their visit to the dental clinic.

**Figure 3:**
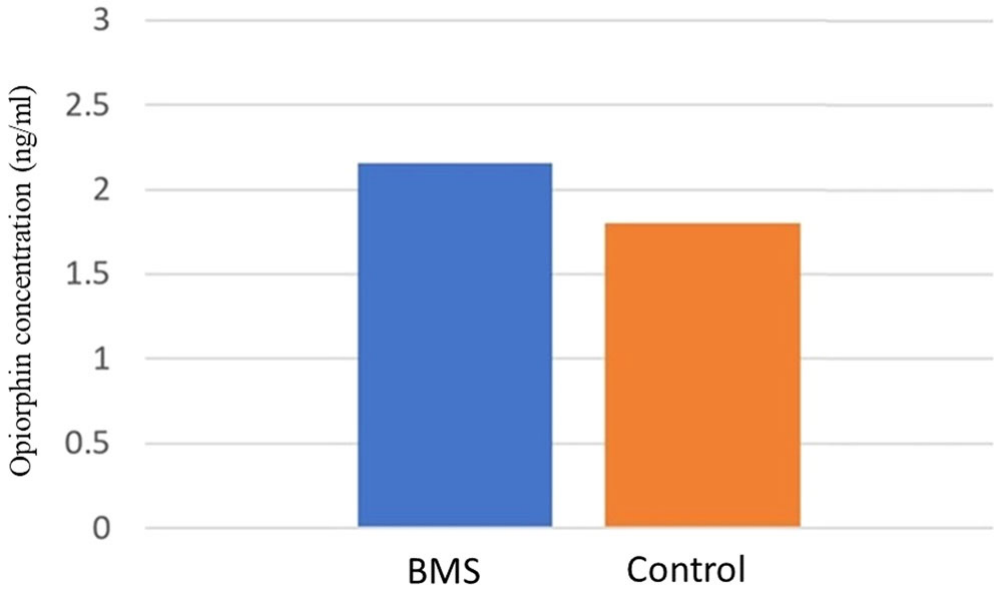
Comparison of opiorphin concentration (ng/mL) between BMS patients and controls.

The mean salivary opiorphin concentration in BMS patients at the beginning of the study was 2.15 ± 0.30 ng/mL. The mean concentration in the control group was 1.80 ± 0.36 ng/mL. The mean opiorphin concentration in BMS patients was significantly higher than that in the controls (*p* < 0.001) (Table 2 and Figure 3).

**Table 2:** Comparison of opiorphin concentration, anxiety, depression, and burning severity between case and control groups and before and after treatment.

	Intergroup			Intragroup		
	BMS group	Control group	*p* value	Before^a^	After	*p* value
VAS, mean ± SD^b^	5.96 ± 1.97	—	—	7.00 ± 2.09	4.18 ± 1.77	< 0.001
Opiorphin concentration (ng/mL)^b^	2.16 ± 0.30	1.80 ± 0.36	< 0.001	2.25 ± 0.15	2.40 ± 0.35	0.212
Anxiety score^b^	12.89 ± 6.27	3.28 ± 3.09	< 0.001	16.63 ± 4.71	12.18 ± 3.97	0.001
Depression score^b^	12.53 ± 4.70	0.92 ± 1.32	< 0.001	12.90 ± 5.43	10.18 ± 4.42	< 0.001

^a^ The information is from 11 patients who attended follow‐up.

^b^ Independent *t*‐test.

The mean BMS anxiety and depression scores at the beginning of the study were 12.89 ± 6.27 and 12.53 ± 4.70, respectively (Table 2). At the first session, 19 subjects had mild anxiety, seven subjects had moderate anxiety, and two subjects had severe anxiety. In addition, three patients had no depression, while 20 patients had mild depression, five patients had moderate depression, and none had severe depression.

Four of the control subjects were excluded from the study due to their anxiety levels. The remaining controls (40 participants) were not diagnosed with anxiety or depression according to the Hamilton questionnaire, with scores below 18 indicating anxiety and scores below six indicating depression.

**Figure 4:**
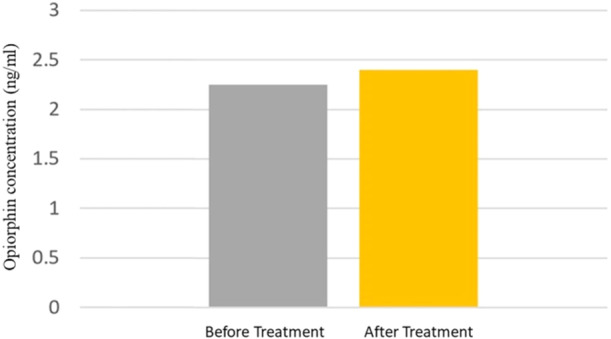
Comparison of opiorphin concentration (ng/mL) in BMS patients before and after treatment.

In addition, 17 patients missed the follow‐up appointment for reasons such as difficulty in commuting, low mood, burning sensation healing, illness, and so forth. Only 11 patients attended follow‐up after 4 weeks; initially, their mean salivary opiorphin concentration was 2.25 ± 0.15 ng/mL, whereas after 4 weeks, their salivary opiorphin concentration was 2.40 ± 0.35 ng/mL, indicating an increase, although insignificant (*p* = 0.212) (Table 2 and Figure 4). However, their initial mean VAS was 7.9 ± 2.09 and it was 4.18 ± 1.77 at the second session, indicating a significant decrease of burning sensation after therapy (*p* < 0.001). Furthermore, the mean anxiety and depression scores were significantly lower in the second session (*p* = 0.001 and *p* < 0.001 for anxiety and depression, respectively).

The correlation between salivary opiorphin levels and age, gender, burning severity, anxiety and depression, stage of menopause, and burning location was not statistically significant (Table 3). However, the mean opiorphin concentration was higher in women and it was higher in postmenopausal women than in other women.

**Table 3:** Correlation between opiorphin concentration and some items in BMS patients and controls.

Groups	BMS group	Control group
*Variables*	*p* value	*p* value
Age	0.138^a^	0.199^a^
Gender	0.727^a^	0.223^a^
VAS	0.552^a^	—
Anxiety score	0.121^a^	0.309^a^
Depression score	0.888^a^	0.908^a^
Duration of disease	0.356^a^	—
Menopause	0.099^b^	0.310^a^
Location	0.897^a^	—

^a^ Pearson correlation coefficient.

^b^ Independent *t*‐test.

## 4 Discussion

In this study, we examined opiorphin concentrations in BMS patients and the association between salivary opiorphin levels and levels of anxiety and depression. We found that almost all BMS subjects showed some level of anxiety and depression. According to the results, initial opiorphin concentrations were significantly higher in the BMS group compared with the controls. Despite the insignificant increase in opiorphin concentrations 4 weeks after treatment, the mean intensity of burning sensation, anxiety, and depression decreased significantly.

Salaric et al. examined BMS and control groups for opiorphin levels of stimulated and unstimulated saliva and reported a significant increase in opiorphin levels in unstimulated saliva of BMS patients. Furthermore, opiorphin levels of stimulated saliva were higher in the BMS group compared with the controls, although the differenced was not significant (Salarić, Sabalić, and Alajbeg 2017).

However, the results are contradictory in the literature. In the study by Boucher et al. the two groups of BMS patients and control subjects did not differ significantly in terms of opiorphin levels in stimulated and unstimulated saliva (Boucher et al. 2017). On the other hand, Ruangsri et al. reported significantly lower unstimulated salivary opiorphin levels in BMS patients (Ruangsri, Jorns, and Chaiyarit 2019), whereas in our study, opiorphin levels were significantly lower in BMS patients compared with the controls.

In Ozdogan et al. opiorphin levels were significantly higher before than after treatment and there was a strong association between pretreatment burning sensation and salivary opiorphin concentrations (Ozdogan et al. 2019). In our study, we found no association between burning severity and opiorphin concentrations. In addition, salivary opiorphin concentrations increased 4 weeks after treatment, although insignificantly. However, because many patients missed follow‐up appointments, it is difficult to interpret and generalize the results.

Such conflicting results suggest that two processes affect opiorphin concentration: (1) since opiorphin reduction plays an important role in neural mechanisms, it might lead to BMS due to disruption of the pain control mechanism, and (2) high opiorphin levels may reflect an adaptive response to chronic pain.

Another reason for higher opiorphin levels in BMS patients is related to encephalin, which acts as a mediator in some diseases including hypertension caused by stress, depression, anxiety, and emotion‐related behaviors, which are common in BMS patients. Therefore, opiorphin can be considered as a marker for the assessment and treatment of conditions that include mood disorders and co‐occurring pain, such as BMS (Javelot et al. 2010).

Brkljačić et al. reported salivary opiorphin levels of 2.8–25.9 ng/mL (Brkljačić et al. 2011). Studies have reported different opiorphin concentrations based on the type of saliva (stimulated or unstimulated), saliva collection method, and the type of kit used.

Salivary opiorphin concentration has been shown to increase in painful conditions, including BMS, through activation of the endogenous analgesic system (Nejad et al. 2020).

In our study, saliva sampling was performed in the morning to eliminate the confounding effect of food intake. However, the severity of burning sensation in the patients was lower in the morning, which could be a reason for the insignificant association between burning severity and opiorphin levels.

Studies have also shown that opiorphin levels are not significantly related to age, gender, menopause stage, burning severity, anxiety, and depression (Boucher et al. 2017, Salarić, Sabalić, and Alajbeg 2017). In our study, the number of postmenopausal women was significantly higher among BMS patients compared with the controls. Similarity, Boucher et al. reported that most BMS patients were postmenopausal women whose anxiety and depression scores were higher than those of healthy individuals (Boucher et al. 2017).

Another cross‐sectional study found that about 73% of BMS patients were female and about 72% were over 50 years of age. They also reported that with each decade of advancing age, the likelihood of BMS in females could increase by more than 3.38 times. In addition, psychological problems increase the susceptibility to BMS in women (Rabiei, Leili, and Alizadeh 2018).

According to studies, BMS patients suffer more often from anxiety and depression disorders (Sikora et al. 2018). Adamo et al. examined 50 BMS patients and concluded that patients suffered from sleep disorders, anxiety, and depression more frequently than controls (Adamo et al. 2013).

In another study by Takao et al. patients with psychiatric disorders complained more often of severe pain (Takao et al. 2023).

In a study conducted in Iran, BMS patients were compared with controls using the Pittsburgh Sleep Quality Index (PSQI) and the Depression, Anxiety, and Stress Scale (DASS‐21). There was a significant association between gender and depression, anxiety, stress, and sleep disorders. Psychiatric problems other than sleep disorders are more common in men (Rezazadeh et al. 2021).

A 2023 meta‐analysis study found that seven types of stress assessment questionnaires were used in the reviewed studies. The results showed that in all studies, BMS patients experienced more stress than controls (Porporatti et al. 2023).

Although anxiety and depression were not included in the inclusion criteria of the present study, almost all BMS patients had some degree of anxiety and/or depression.

Boucher et al. examined anxiety and depression using the HAD‐A and HAD‐D questionnaires and found no association between the severity of anxiety and depression and opiorphin concentrations. They hypothesized that patients may be predisposed to acute stress due to anxiety and depression and that increased opiorphin may be due to acute daily stress (Boucher et al. 2017).

On the other hand, given the antidepressant effect of opiorphin (Javelot et al. 2010), the association between higher concentrations of opiorphin after treatment and lower levels of anxiety and depression could be justified.

A limitation of the present study was the small number of patients for posttreatment assessment, making it difficult to evaluate the effectiveness of psychiatric treatment.

Further studies are required to confirm the association between opiorphin concentration and burning sensation severity in BMS patients. Determination of salivary opiorphin concentration may be useful as a noninvasive method for assessing the effectiveness of treatment. It is recommended that opiorphin concentrations be assessed concurrently with assessment of BMS symptom severity, depression, and anxiety during long‐term follow‐up.

## Author Contributions

Study conceptualization: Zohreh Dalirsani and Maryam Amirchaghmaghi. Data handling: Mahshid Malakuti Semnani and Ava Taghizadeh. Experimental design: Zohreh Dalirsani, Maryam Amirchaghmaghi, Mahdi Talebi, and Seyed Isaac Hashemy. Analysis and interpretation of data: Mohammad Taghi Shakeri. Provision of study materials and equipment: Mahshid Malakuti Semnani Ava Taghizadeh and Seyed Isaac Hashemy. Study validation: Mohammad Taghi Shakeri and Mahdi Talebi. Supervision: Maryam Amirchaghmaghi, Zohreh Dalirsani, and Mahdi Talebi. Data presentation: Mahshid Malakuti Semnani and Mohammad Taghi Shakeri. Draft preparation: Maryam Amirchaghmaghi, Zohreh Dalirsani, and Mahshid Malakuti Semnani. Study consultation: Zohreh Dalirsani and Mahdi Talebi. Writing and reviewing: Maryam Amirchaghmaghi, Zohreh Dalirsani, and Mahshid Malakuti Semnani. Project administration: Zohreh Dalirsani and Maryam Amirchaghmaghi. All authors approved the final version of this article.

## Ethics Statement

This study was approved by the Ethics Committee of Mashhad University of Medical Sciences under the code IR.MUMS.DENTISTRY.REC.1397.045.

## Conflicts of Interest

The authors declare no conflicts of interest.

## Data Availability

The data sets generated during and/or analyzed during the current study are available from the corresponding author on reasonable request.
